# A Network of Multiple Regulatory Layers Shapes Gene Expression in Fission Yeast

**DOI:** 10.1016/j.molcel.2007.03.002

**Published:** 2007-04-13

**Authors:** Daniel H. Lackner, Traude H. Beilharz, Samuel Marguerat, Juan Mata, Stephen Watt, Falk Schubert, Thomas Preiss, Jürg Bähler

**Affiliations:** 1 Cancer Research UK Fission Yeast Functional Genomics Group, Wellcome Trust Sanger Institute, Hinxton, Cambridge CB10 1HH, UK; 2 Molecular Genetics Program, Victor Chang Cardiac Research Institute, Darlinghurst, NSW 2010, Australia; 3 St Vincent's Clinical School and School of Biotechnology and Biomolecular Sciences, University of New South Wales, Sydney, NSW 2052, Australia

**Keywords:** RNA

## Abstract

Gene expression is controlled at multiple layers, and cells may integrate different regulatory steps for coherent production of proper protein levels. We applied various microarray-based approaches to determine key gene-expression intermediates in exponentially growing fission yeast, providing genome-wide data for translational profiles, mRNA steady-state levels, polyadenylation profiles, start-codon sequence context, mRNA half-lives, and RNA polymerase II occupancy. We uncovered widespread and unexpected relationships between distinct aspects of gene expression. Translation and polyadenylation are aligned on a global scale with both the lengths and levels of mRNAs: efficiently translated mRNAs have longer poly(A) tails and are shorter, more stable, and more efficiently transcribed on average. Transcription and translation may be independently but congruently optimized to streamline protein production. These rich data sets, all acquired under a standardized condition, reveal a substantial coordination between regulatory layers and provide a basis for a systems-level understanding of multilayered gene-expression programs.

## Introduction

The characteristics of organisms result largely from the complex interplay between DNA or RNA and the regulatory apparatus. Proper control of gene expression is fundamental to implement the information in the genome and pervades most of biology, from cell proliferation and differentiation to development. Gene expression is controlled at multiple levels, and cells need to coordinate different regulatory processes to function properly and prevent disease. Protein production is influenced by transcription rate, mRNA polyadenylation and stability, and translation rate among other factors. There is increasing appreciation that the different processes involved in gene expression are integrated with each other (Maniatis and Reed, 2002; Moore, 2005; Orphanides and Reinberg, 2002; Proudfoot et al., 2002). The sophistication of gene-expression control has been recognized through numerous in-depth studies on the regulation of specific genes at several levels (Lal et al. [2006] and references therein).

Large-scale approaches provide powerful complementary insight into regulatory mechanisms from a global perspective. Microarrays have been widely used to measure mRNA steady-state levels for expression profiling (Lockhart and Winzeler, 2000). More recently, variations of microarray methods have been applied to measure additional aspects of gene expression (Hieronymus and Silver, 2004; Mata et al., 2005). These elegant approaches have mostly been pioneered in the budding yeast *Saccharomyces cerevisiae*: examples include genome-wide studies on mRNA half-lives (Grigull et al., 2004; Wang et al., 2002), RNA binding proteins (Gerber et al., 2004), and translation (Arava et al., 2003; MacKay et al., 2004; Preiss et al., 2003). These global data sets provide supplementary and unique views on specific aspects of gene expression and allow the discovery of unexpected connections.

Whereas traditional studies address multiple aspects of regulation for one or a few genes, genome-wide studies typically are restricted to one aspect of regulation. It is not clear to what degree different regulatory levels of gene expression are coordinated with each other at a global scale to optimize protein production. A comprehensive understanding of gene expression will require integrated genome-wide data covering multiple regulatory intermediates, given that the cell itself regulates and coordinates multiple levels of gene expression.

Here, we present genome-wide data sets for key aspects of gene expression in the fission yeast *Schizosaccharomyces pombe.* A detailed analysis of global translational properties is complemented by several other large-scale data for context and comparisons. The integrated analyses further incorporated publicly available information on *S. pombe* ORF lengths (Wood et al., 2002) and protein levels (Matsuyama et al., 2006). This multidimensional approach provides broad coverage of gene-expression intermediates by using a standardized growth condition and coherent methodology. The systematic and quantitative data sets helped to uncover global connections and trends that would not be apparent from studies involving only a few genes, and they revealed remarkably widespread relationships between multiple layers of gene expression.

## Results

### Global Translational Properties of mRNAs

**Figure 1:**
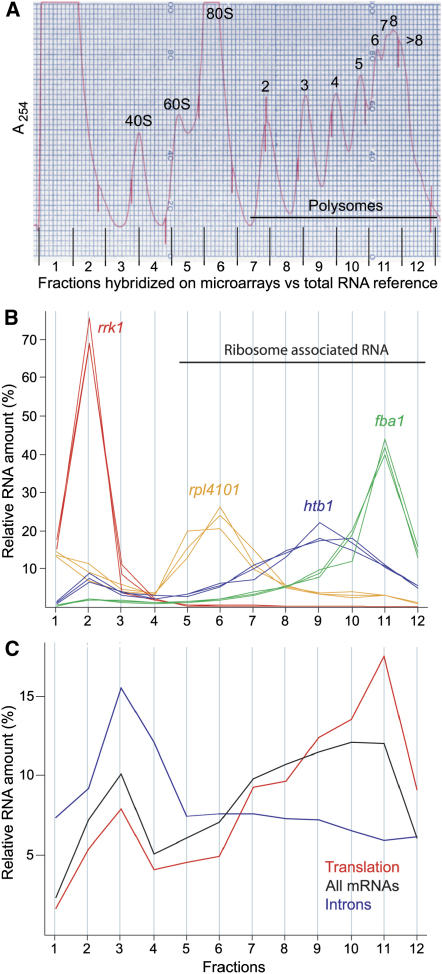
Genome-Wide Translational Profiling (A) Polysome profile showing the absorbance of RNA separated by density on a sucrose gradient, reflecting the number of associated ribosomes. The peaks of the profile are labeled for the small and large ribosomal subunits (40S and 60S), the monosome (80S), and the numbers of associated ribosomes for polysomal RNA (2 to >8). RNA from 12 fractions equally spaced throughout the profile (bottom) was labeled and hybridized against a total RNA reference on microarrays containing all *S. pombe* genes. (B) Translation profiles for selected transcripts obtained by microarray analysis, showing the relative RNA amounts for a given transcript contained in each of the 12 fractions. Fractions associated with ribosomes are indicated. Different transcripts are color coded, and polysome profiles from three independent biological repeats are shown for *rrk1* (RNase P K-RNA), *rpl4101* (encoding ribosomal protein), *htb1* (encoding histone H2B), and *fba1* (encoding fructose-biphosphate aldolase). (C) Average translation profiles for selected groups of RNAs, plotted as in (B) for one experiment. All mRNAs, the 3505 high-confidence mRNAs with complete profiles in this experiment; Introns, 11 long introns included on the microarray; and Translation, 62 mRNAs associated with the GO terms “translational intiation,” “translational elongation,” or “translational termination.”

To obtain high-resolution translational data for vegetatively growing *S. pombe* cells at a genome-wide scale, we fractionated polysomes and interrogated microarrays with RNA fractions representing different numbers of associated ribosomes (Figure 1A). Figure 1B provides examples of translation profiles from three independently repeated experiments, showing high reproducibility between experiments. We verified that transcripts peaked in the expected fractions. For instance, the noncoding *rrk1* RNA peaked in fraction 2, reflecting an absence of associated ribosomes as expected for an RNA that is not translated. The *fba1* mRNA peaked in fraction 11, reflecting an association with many ribosomes for most of the mRNA; consistent with this, Fba1p is highly expressed and within the top 1% with respect to protein levels (Hwang et al., 2006). The 78 nucleotide *rpl4101* is the shortest mRNA in *S. pombe* and is therefore not expected to be associated with many ribosomes; accordingly, it peaked around fraction 6, which corresponds to the binding of a single ribosome. These profiles obtained by microarrays corresponded well with independent profiles obtained by quantitative PCR (Bachand et al., 2006; data not shown).

Figure 1C shows average translational profiles for selected groups of transcripts. The profile of all mRNAs that provided translational data peaked in fraction 3 (reflecting free mRNA) along with a broad peak covering fractions 7–11 (reflecting polysomes of different sizes). Introns that were included on the microarrays peaked in fraction 3, as expected given that translation occurs on spliced mRNA. Conversely, mRNAs associated with Gene Ontology (GO) terms for translational regulation were associated with many ribosomes as expected for these highly expressed genes. A group of 481 mRNAs encoding secreted proteins that are translated on the endoplasmic reticulum showed an almost identical average translational profile to the one for all mRNAs (data not shown), indicating a similar ribosome distribution for this specialized group.

Although polysome profiles were obtained for almost all mRNAs, we focused on a conservative, high-confidence set of 3598 (72.5%) out of the 4962 nuclear protein-coding genes. Most of the excluded genes were not or only weakly expressed under the condition used (Figure S1 in the Supplemental Data available with this article online) and were most enriched for GO terms related to meiosis (p < 4e^−24^). From the translation profiles, we determined different properties reflecting translational efficiency as described below with corresponding data provided in Table S1.

Ribosome occupancy indicates the percentage of a given type of mRNA that is associated with one or more ribosomes. The average ribosome occupancy was 77.3% with a relatively small standard deviation (SD) of 7.0%. This suggests that during exponential growth the majority of high-confidence mRNAs are engaged in translation, although a substantial fraction (>20%) of mRNAs is not associated with any ribosomes.

The mean number of ribosomes bound to a given mRNA was calculated based on a weighted average by using the relative amount of the mRNA associated with each fraction and the number of ribosomes corresponding to that fraction. Only fractions associated with ribosomes were included so that the mean ribosome number is independent of ribosome occupancy. On average, 4.1 ribosomes were associated with mRNAs with a surprisingly small SD of 0.6. If the mRNAs not associated with ribosomes were also taken into account, this value was lowered to 3.6 ribosomes. As expected, the mean number of associated ribosomes generally increased as a function of open reading frame (ORF) length (Figure S2).

Arguably, the ribosome density is a better measure than the mean ribosome number to estimate translational efficiency as it normalizes for different mRNA lengths that influence the numbers of bound ribosomes (Figure S2) (Arava et al., 2003; Beyer et al., 2004). Overall, mRNAs showed a mean ribosome density of 4.5 ribosomes/kilobase (kb) of ORF, with a large SD of 3.1 ribosomes/kb. On average, the mRNAs thus contained one ribosome every ∼222 nucleotides. Given that a eukaryotic ribosome occupies ∼35 nucleotides of mRNA (Wolin and Walter, 1988), the average density determined here is only ∼1/6 of the maximal packing density. This is consistent with initiation being the rate-limiting factor for translation.

The sequence context of the AUG start codon influences the rate of translational initiation (Kozak, 1991). To corroborate that high ribosome occupancy and density in our data reflect efficient translational initiation rather than slow elongation or ribosome stalling, we determined the “AUG context adaptation index” (AugCAI), a measure for the effectiveness of the AUG context to promote translational initiation (Miyasaka, 2002). This analysis provided a consensus sequence for optimal translational initiation in *S. pombe* and revealed significant correlations of the AugCAI with ribosome occupancy and density (Figure S3). This provides independent evidence that the translational profiling data are measures for translational efficiency.

We next looked for highly and poorly translated mRNAs. The 20% of mRNAs with the highest ribosome occupancy were most enriched for transcripts repressed during stress, many of which are involved in protein synthesis (p ∼ 8e^−30^ [Chen et al., 2003]) and for those associated with the GO terms “metabolism” and “biosynthesis” (p < 1e^−30^). The 20% of mRNAs with the lowest ribosome occupancy were diverse and showed no strong enrichment for any particular GO terms or functional groups. The 20% of mRNAs with the highest ribosome density were most enriched for GO terms such as “ribosome,” “organelle,” and terms related to mitochondria (p < 1e^−12^) and for transcripts containing introns (p ∼ 5e^−17^), which is notable given that introns can enhance translation in mammals (Nott et al., 2003). The 20% of mRNAs with the lowest ribosome density were most enriched for the GO terms “ATP binding,” “hydrolase activity,” “signal transduction,” and related terms (p < 2e^−10^). The mRNAs with low ribosome density were also strongly enriched for the longest mRNAs, whereas those with high ribosome density were enriched for the shortest mRNAs. This suggested a connection between mRNA length and ribosome density as described below.

### Short mRNAs Are More Efficiently Translated

**Figure 2:**
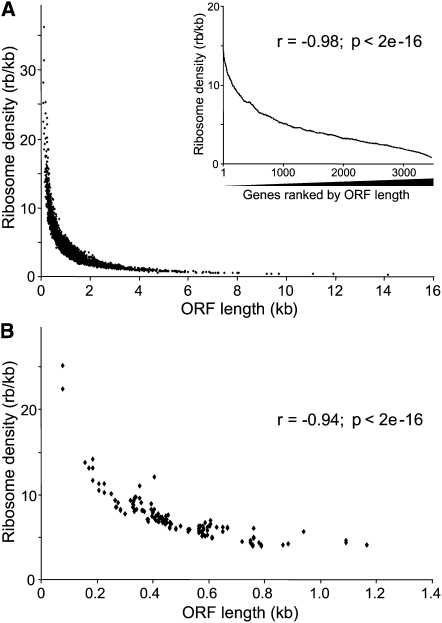
Inverse Correlation between Ribosome Density and ORF Length (A) Ribosome density plotted against ORF length for the 3598 high-confidence mRNAs. The inset graph shows moving averages (100 gene window) of ribosome density as a function of genes ranked by ORF length. The corresponding Spearman rank correlation is also shown. (B) Ribosome density plotted against ORF length as in (A) but showing only the 134 mRNAs encoding ribosomal proteins, along with corresponding Spearman rank correlation.

Whereas the mean ribosome numbers varied <4-fold (1.8–6.8 ribosomes/mRNA), the ORF lengths varied >180-fold (78–14,154 bp). Accordingly, the ribosome numbers showed only modest increase relative to ORF length and did not increase above ∼4.3 ribosomes on average for mRNAs longer than ∼1200 bp (Figure S2). These observations indicate that ORF length is a major factor determining ribosome density. There was indeed a strong inverse correlation between ORF length and ribosome density (Figure 2A). Short mRNAs were much more tightly packed with ribosomes than long mRNAs. This inverse correlation was evident over the whole range of ORF sizes and ribosome densities. A similar inverse correlation was obtained when using mRNA lengths instead of ORF lengths based on 198 mRNAs for which untranslated regions (UTRs) are available from *S. pombe* GeneDB (r = −0.9; p < 2e^−16^).

We wondered whether this inverse correlation might reflect a systematic artifact of translational profiling. A bias could arise from underestimating the numbers of ribosomes in the poorly resolved higher fractions where single-peak resolution cannot be achieved (Figure 1A). We observed a similar negative correlation, however, when using only the relatively short mRNAs encoding ribosomal proteins (Figure 2B and Figure S2); these mRNAs showed defined peaks in the well-resolved fractions of the polysome profiles where ribosome numbers can be determined with confidence (Figure 1A, fractions 6–10). To further exclude a possible error due to underestimating ribosomes, we associated double the originally estimated number of ribosomes with fraction 12. This reanalysis resulted in a similar negative correlation between ribosome density and ORF length (Figure S4).

**Figure 3:**
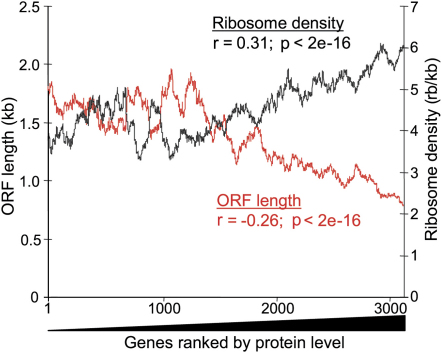
Correlations between ORF Length and Ribosome Density with Protein Level Moving averages (100 gene window) of ribosome density (black) and ORF length (red) as a function of 3265 genes ranked by protein level. The Spearman rank correlations between protein level and ribosome density (n = 3265) and between protein level and ORF length (n = 4434) are also shown.

We also observed a significant inverse correlation between ORF length and ribosome occupancy, although much less pronounced than for ribosome density (r = −0.27; p < 2e^−16^). Moreover, the AugCAI showed an inverse relationship with ORF length (r = −0.15; p < 2e^−16^), providing independent evidence for a link between ORF length and translational efficiency. We therefore expected long proteins to be present in lower levels than short proteins due to differences in translational efficiency. To test this hypothesis, we took advantage of global data on *S. pombe* protein expression levels (Matsuyama et al., 2006), which were obtained after integrating all ORFs into the same genomic site and transcribing them from the same promoter. These data should therefore be minimally affected by differences in transcription or posttranscriptional regulation via UTR sequences, as the lengths and sequences of the ORFs are the only remaining factors that could influence translational efficiency, which (along with protein turnover) will determine protein levels. The ribosome densities showed a significant positive correlation with protein levels, whereas ORF length negatively correlated with protein levels as predicted from our translational profiling data (Figure 3). For proteins present at lower levels, the correlations with ribosome density and ORF length were less evident, possibly due to increased noise. The protein levels also correlated with ribosome occupancy (r = 0.31; p < 2e^−16^). We conclude that ORF length directly or indirectly affects translational efficiency and protein levels.

### mRNAs with Long Poly(A) Tails Are More Efficiently Translated

The 3′-poly(A) tails of mRNAs are thought to determine the efficiency of translational initiation based on single-gene studies (Wickens et al., 2000). We therefore wondered whether translational efficiency might be reflected in poly(A) tail lengths on a genome-wide scale. To obtain global data on polyadenylation, we fractionated the mRNAs with a poly-U Sepharose column followed by differential elution at five temperatures. Five mRNA fractions with distinct ranges of poly(A) tail length were then hybridized to microarrays with total eluate as a reference. This approach, termed polyadenylation state array (PASTA) analysis, will be described in more detail elsewhere (T.H.B and T.P., unpublished data). The fractions contained distinct but partially overlapping distributions of poly(A) tail lengths, ranging from ∼10 to 80 nucleotides (Figure S5). These data revealed a continuous distribution of poly(A) tail lengths, both for specific mRNAs and between different mRNAs. Nevertheless, the poly(A) profiles for different mRNAs were enriched for distinct sizes.

We used a modified RT-PCR assay, termed ligation-mediated poly(A) test (LM-PAT) (Sallés and Strickland, 1995), to verify the poly(A) profiles derived from the PASTA analysis. The poly(A) tails of mRNAs with different tail-length distributions showed good agreement between PASTA analysis and LM-PAT-assays (Figure S6). Moreover, mitochondrially encoded mRNAs, which lack poly(A) tails in fission yeast (Schäfer et al., 2005), showed the expected peak in the first fraction.

We ranked the mRNAs by relative poly(A) tail length using a weighted average of the relative amounts of mRNA associated with each fraction (Table S1). The 20% of mRNAs with the longest tails were most enriched for transcripts repressed during stress (p ∼ 1e^−15^ [Chen et al., 2003]) and for the GO terms “biosynthesis,” “cytoplasm,” and “ribosome” (p < 2e^−16^). The 20% of mRNAs with the shortest tails were most enriched for genes containing predicted nuclear localization signals (p ∼ 1e^−18^) and for the GO terms “nuclear lumen,” “nucleolus,” “RNA metabolism,” and “ribosome biogenesis and assembly” (p < 3e^−8^).

**Figure 4:**
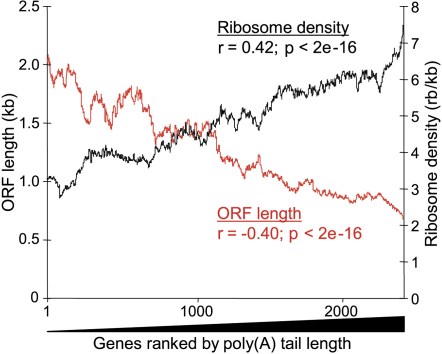
Correlations between ORF Length and Ribosome Density with Poly(A) Tail Length Moving averages (100 gene window) of ribosome density (black) and ORF length (red) as a function of 2576 genes ranked by poly(A) tail length. The Spearman rank correlations between poly(A) tail length and ribosome density (n = 2576) and between poly(A) tail length and ORF length (n = 2714) are also shown.

Poly(A) tail lengths significantly increased with increasing ribosome density (Figure 4). Accordingly, poly(A) tail lengths increased with decreasing ORF lengths (Figure 4), consistent with the strong inverse correlation between ORF length and ribosome density (Figure 2). These data were corroborated by genome-wide binding data for the poly(A) binding protein Pab1p: Pab1p was most enriched in precipitated mRNAs with long poly(A) tails, and Pab1p enrichment showed a strong inverse correlation with ORF lengths (J.M., unpublished data). Poly(A) tail lengths also correlated with ribosome occupancy (r = 0.27; p < 2e^−16^) and with protein levels (r = 0.21; p < 2e^−16^). Together, these data reveal a genome-wide connection between ORF length, poly(A) tail length, and translational efficiency: short mRNAs tend to have long poly(A) tails and are more efficiently translated than longer mRNAs that tend to have shorter poly(A) tails. These connections are ultimately reflected at the protein levels and are most evident for the highly expressed proteins (Figure 3).

### Abundant mRNAs Are More Efficiently Translated

**Figure 5:**
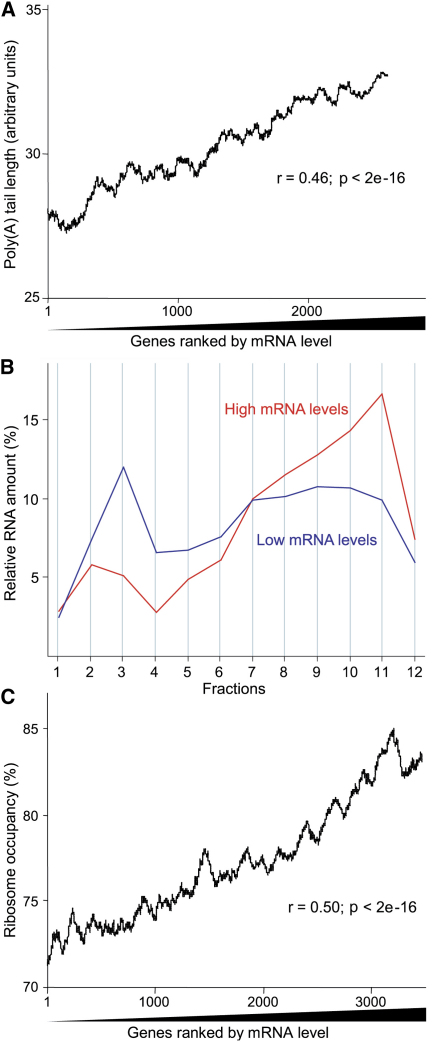
Correlations between mRNA Level and Poly(A) Tail Length and Ribosome Occupancy (A) Moving averages (100 gene window) of poly(A) tail length as a function of 2688 genes ranked by mRNA level, along with corresponding Spearman rank correlation. (B) Average translation profiles of the mRNAs with the 500 highest (red) or 500 lowest (blue) levels plotted as in Figure 1B. (C) Moving averages (100 gene window) of ribosome occupancy as a function of 3567 genes ranked by mRNA level, along with corresponding Spearman rank correlation.

Steady-state mRNA levels are another important determinant of gene expression. We estimated the mRNA levels in exponentially growing cells from the hybridization signal intensities by using Affymetrix chips (Table S1). These data were in good agreement with independent data for mRNA levels obtained by hybridizing mRNA against a genomic DNA reference on our in-house DNA microarrays (data not shown). The 10% most abundant mRNAs were most enriched for transcripts repressed during environmental stress (p ∼ 2e^−86^ [Chen et al., 2003]) and for the GO terms “ribosome,” “protein biosynthesis,” “cellular metabolism,” and related terms (p < 2e^−55^). The 10% least abundant mRNAs were most enriched for transcripts induced during meiosis and stress (p < 3e^−15^ [Mata et al., 2002; Chen et al., 2003]), for *S. pombe-*specific transcripts (p ∼ 1e^−34^ [Mata and Bähler, 2003]), and for GO terms such as “meiosis” and “M phase” (p < 1e^−18^). The mRNA levels did not correlate with ORF lengths (Figure S7). They significantly correlated, however, with poly(A) tail lengths: the most abundant mRNAs showed a tendency for longer tails (Figure 5A).

We then checked for relationships between mRNA levels and translational efficiency. The mRNAs with the lowest expression levels tended to be associated with fewer ribosomes than the mRNAs with the highest levels (Figure 5B). This raised the possibility that mRNA abundance is somehow coordinated with translational efficiency. Consistent with this, ribosome densities showed some correlation with mRNA levels (r = 0.14; p < 2e^−16^). Stronger correlations throughout the entire population of mRNAs were apparent between ribosome occupancies and mRNA levels (Figure 5C). The AugCAI also significantly correlated with mRNA levels (r = 0.22; p < 2e^−16^). Taken together, these findings indicate a genome-wide coordination between mRNA level and translational efficiency: more abundant mRNAs tend to be more efficiently translated as reflected by their higher ribosome occupancy and, to a lesser extent, higher ribosome density.

### Stable and Highly Transcribed mRNAs Are More Efficiently Translated

The steady-state level of a given mRNA is determined by the rate of transcription and the rate of decay, both of which are controlled at genome-wide levels (Mata et al., 2005). The correlation between translational efficiency and mRNA abundance could therefore reflect a connection between translation and mRNA stability and/or between translation and transcription.

**Figure 6:**
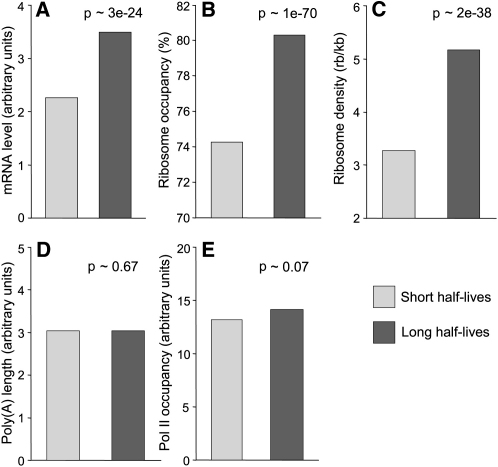
Correlations between mRNA Half-Lives and Other Gene Expression Properties Bar graphs showing the mean mRNA levels (A), ribosome occupancies (B), ribosome densities (C), poly(A) tail lengths (D), and Pol II occupancies (E) for two groups of mRNAs with either short (light gray) or long (dark gray) half-lives. The significance of the difference between the means from the two mRNA groups is given for each panel.

Abundant mRNAs are expected to be more stable on average than less abundant mRNAs. To test whether mRNA stability is linked to translation, we estimated global mRNA half-lives by blocking transcription and measuring mRNA levels at different times after transcriptional shut off (Figure S8 and Table S1). These experiments provided reliable estimates on relative half-lives for the 868 least-stable mRNAs, with half-lives ranging from ∼10 to 96 min and a median of ∼33 min. This group of unstable mRNAs was enriched for genes with periodic expression during the cell cycle (p ∼ 6e^−15^; Marguerat et al., 2006; Rustici et al., 2004); these mRNAs peak in levels during a short cell-cycle phase and are therefore expected to have short half-lives. The unstable mRNAs were also enriched for genes associated with the GO terms “regulation of biological process,” “cell communication,” “signal transduction,” and “cell septum” (p < 1e^−5^). This probably reflects that mRNAs encoding regulatory proteins or proteins only required during a defined stage such as septation need to be tightly controlled. We also selected a similarly sized group of bona fide stable mRNAs whose expression levels were not altered 30 min after transcriptional shut off (Figure S8). This group was most enriched for genes with the GO terms “cytoplasm” and “mitochondrial part” (p < 5e^−5^). As expected, mRNAs with short half-lives were significantly less abundant on average than mRNAs with longer half-lives (Figure 6A).

We then checked for relationships between mRNA stability and translational efficiency. The mRNAs with long half-lives showed significantly higher ribosome occupancies and densities on average than mRNAs with short half-lives (Figures 6B and 6C). Thus, efficiently translated mRNAs tend to be more stable than less efficiently translated mRNAs. Although translational efficiency correlated with both poly(A) tail length and mRNA stability, we did not detect any correlation between mRNA stability and poly(A) tail length (Figure 6D).

**Figure 7:**
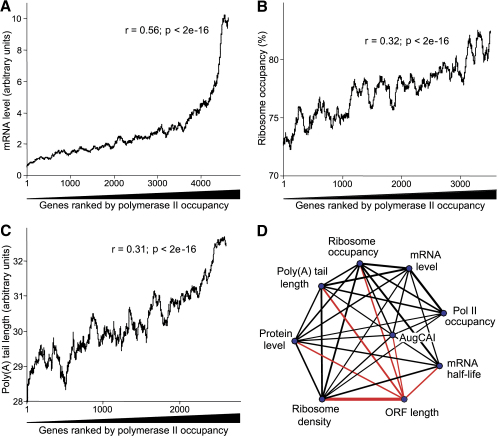
Correlations between Pol II Occupancy and Other Gene Expression Properties, and Relationships between All Studied Properties (A) Moving averages (100 gene window) of relative mRNA level as a function of 4724 genes ranked by Pol II occupancy, along with corresponding Spearman rank correlation. (B) Moving averages (100 gene window) of ribosome occupancy as a function of 3598 genes ranked by Pol II occupancy, along with corresponding Spearman rank correlation. (C) Moving averages (100 gene window) of poly(A) tail length as a function of 2713 genes ranked by Pol II occupancy, along with corresponding Spearman rank correlation. (D) Weighted association map summarizing the relationships between all aspects of gene expression analyzed here. The blue nodes represent the different data sets as labeled, black lines show significant positive correlations between the connected data sets, and red lines show significant inverse correlations. The weight of the lines reflects the absolute correlation value.

Besides mRNA stability, does transcription also contribute to the correlation between mRNA levels and translation? The relative amount of RNA polymerase II (Pol II) associated with a given ORF provides an estimate for transcriptional efficiency (Sandoval et al., 2004). We therefore established a systematic approach to measure Pol II occupancy by using chromatin immunoprecipitation followed by analysis on microarrays (Table S1). The 10% of genes that were either most or least associated with Pol II showed similar enrichments for GO terms and functional groups as the 10% most or least abundant mRNAs, respectively. The mitochondrially encoded genes were a notable exception; they showed high mRNA levels but were strongly underenriched in the Pol II precipitations, consistent with these genes being transcribed by a different RNA polymerase (Schäfer et al., 2005). Transcriptional efficiency did not significantly correlate with mRNA stability (Figure 6E), but it correlated with mRNA levels as expected (Figure 7A).

We next checked for relationships between transcriptional and translational efficiencies. Pol II occupancy showed a correlation with ribosome occupancy (Figure 7B) and a marginal, albeit significant, correlation with ribosome density (r = 0.11; p ∼ 3e^−11^). Thus, both transcription and mRNA turnover are reflected at the level of translation: efficiently transcribed and stable mRNAs tend to be more efficiently translated.

Surprisingly, transcriptional efficiency also correlated with poly(A) tail lengths (Figure 7C). This is in contrast to the apparent absence of any connection between mRNA stability and poly(A) tails (Figure 6D) but is consistent with the correlation between mRNA levels and poly(A) tails (Figure 5A). It was tempting to hypothesize that poly(A) tail lengths are determined by transcription rates. To test this idea, we analyzed polyadenylation for specific mRNAs that were transcribed at different rates by using regulatable promoters (Figure S9). This analysis indicates that the transcription rate does not influence poly(A) tail length. When transcription was induced within a short time, however, a transient population of longer tailed mRNAs was apparent, which were then deadenylated with different kinetics depending on the particular mRNA (Figure S9). We conclude that the transcription rate does not directly influence poly(A) tail lengths, although increased transcription can lead to transiently increased tail lengths before reaching steady-state conditions.

## Discussion

Our translational profiling analysis gives comprehensive insight into translational properties for most mRNAs of fission yeast, thus providing different measures for translational efficiency. The 20% of mRNAs with the lowest ribosome densities significantly overlapped with a list of orthologous genes being poorly translated in budding yeast (p ∼ 5e^−9^ [Law et al., 2005]). This indicates that translational efficiency for a substantial number of mRNAs is conserved across evolution. Overall, our numbers of bound ribosomes and average ribosome density are ∼30% lower than those previously reported for budding yeast, while the ribosome occupancies are similar (this study; Arava et al., 2003). Some of this discrepancy could reflect differences in calculating ribosome numbers. Moreover, fission yeast was cultured in minimal medium, whereas budding yeast was cultured in rich medium that allows faster growth and presumably higher translational efficiency. In addition, fission yeast grows ∼30% slower than budding yeast even in rich medium, and it is therefore possible that this growth difference is reflected (or even driven) by a generally higher translational efficiency in the latter.

To uncover global relationships between translational efficiency and other properties and intermediates of gene expression, we have acquired complementary genome-wide data on transcriptional efficiency and on mRNA polyadenylation, abundance, and stability in *S. pombe* cells grown under the standardized condition used for translational profiling. These data have then been put in context with each other and with data on ORF length, AugCAI index, and protein levels. This analysis reveals an extensive coordination between different aspects of gene expression. Figure 7D summarizes the widespread correlations between the independent data sets, highlighting a complex interplay between multiple gene expression layers.

We have identified two basic properties of mRNAs that are coordinated with translational efficiency: length and abundance. Translation tends to be more efficient for shorter and more abundant mRNAs. Shorter and more abundant mRNAs also tend to have longer poly(A) tails, in accordance with small-scale data indicating that poly(A) tail length influences translational efficiency or vice versa (Preiss et al., 1998; Schwartz and Parker, 1999; Wickens et al., 2000). Thus, mRNA length and abundance are aligned on a genome-wide scale with both poly(A) tail length and translational efficiency. The lengths and levels of mRNAs, however, show no correlation with each other (Figure S7), suggesting that these two mRNA properties are connected with translation independently of each other. Notably, mRNA lengths correlate most with ribosome density (Figure 2), whereas mRNA levels correlate most with ribosome occupancy (Figure 5C). These two measures of translational efficiency may reflect distinct and partially independent mechanisms of translational control.

The poly(A) tail-length distribution of the budding yeast transcriptome has been surveyed in a similar way as reported here (T.H.B and T.P., unpublished data). In both yeasts, the mRNAs with long tails are enriched for ribosomal proteins, whereas the mRNAs with short tails are enriched for ribosomal biogenesis functions. Thus, these two related functional groups can be distinguished based on their poly(A) tail-length distributions. Another similarity is that long-tailed mRNAs are enriched for cytoplasmic functions while short-tailed mRNAs are enriched for nuclear functions. The evolutionary conservation of these features suggests that poly(A) tail lengths have functional importance, or it could reflect conserved regulation at other levels (e.g., the efficiency of translation may affect deadenylation [Schwartz and Parker, 1999]). Comparisons of overall polyadenylation reveal that the distribution of the poly(A) tail profile tends toward longer tails in *S. pombe*, whereas the maximal length is similar between the two yeasts (Figure S5). We speculate that this might be due to the absence of cytoplasmic poly(A) adenylases in budding yeast (Stevenson and Norbury, 2006); these enzymes may readenylate some short-tailed transcripts in fission yeast.

### mRNA Length and Translational Efficiency

Available data suggest that the relationship between mRNA length and translational efficiency is conserved during evolution. Synonymous codon usage, which is thought to affect the accuracy or rate of translation, and the AugCAI index are both negatively correlated with gene length in multicellular eukaryotes (Duret and Mouchiroud, 1999; Miyasaka, 2002). In budding yeast, mRNA length and protein size are inversely correlated with ribosome density and protein levels, respectively (Arava et al., 2003; Warringer and Blomberg, 2006), and proteins present at high copies per mRNA tend to be of low molecular weight (Lu et al., 2007). We find that mRNA length is inversely correlated with several independent measures for translational efficiency such as ribosome density and occupancy, AugCAI, poly(A) tail length, mRNA half-life, and protein level (Figure 7D).

It is not clear why mRNA length and translational efficiency are linked. Ribosome-density mapping for specific mRNA portions has indicated that differences in translation initiation, rather than elongation or termination, determine ribosome densities in mRNAs of different lengths (Arava et al., 2005). Why would initiation of translation be more efficient for shorter mRNAs? One possibility is simply a higher likelihood for the formation of complex secondary structures in longer mRNAs that could inhibit initiation (Hershey and Merrick, 2000). Alternatively or in addition, the mRNA closed-loop model (Kahvejian et al., 2001) suggests that interaction between the 5′UTR and the 3′-poly(A) tail is important for initiation, and it could be easier for shorter mRNAs to achieve this conformation. It is well possible, however, that the mRNA length has no direct influence on translational efficiency but is an independently co-opted parameter (see below).

Integration of our findings with *S. pombe* ORFeome data (Matsuyama et al., 2006) show that the inverse relationship between ORF length and translational efficiency is ultimately reflected in protein levels: longer mRNAs tend to encode less abundant proteins. It is possible that this tendency reflects cellular parsimony. The synthesis of longer proteins is energetically more costly, and there could be evolutionary pressure for abundant proteins to become smaller. The highly abundant ribosomal proteins, for example, are all relatively small. Thus, mRNA length may be a co-opted parameter reflecting an overall goal for gene expression but without any direct mechanistic link to polyadenylation and translation. Notably, the tendency of short mRNAs to be highly expressed is only implemented at the translational level; no correlations between ORF length and transcription or mRNA levels are evident from our data (Figure 7D).

### mRNA Abundance and Translational Efficiency

Unlike mRNA length, mRNA abundance positively correlates with translational efficiency and with related but independent measures such as AugCAI, poly(A) tails, and protein levels (Figure 7D). Transcriptional efficiency and mRNA half-lives contribute to mRNA steady-state levels, and both of them also seem to contribute to the link between mRNA levels and translational efficiency, as they both correlate with translational efficiency and related measures (Figure 7D). Although both transcriptional efficiency and mRNA half-lives correlate with mRNA levels, they do not correlate with each other (Figure 6E), suggesting that they are independently coordinated with translation. The connection between mRNA half-lives and translational efficiency is not unexpected given that translation inhibits mRNA decay (Parker and Song, 2004). Our data indicate that more efficiently translated mRNAs are better protected from decay. Consistent with this model, mRNA half-lives correlate positively with the AugCAI and with protein levels and negatively with ORF length (Figure 7D). A global study on mRNA decay in budding yeast, however, did not detect correlations between mRNA half-lives and mRNA levels or ribosome densities (Wang et al., 2002). We speculate that this discrepancy reflects differences in methodology rather than biological differences between the two yeasts.

The half-lives of mRNAs do not correlate with poly(A) tail lengths. Deadenylation is required for mRNA decay (Parker and Song, 2004), but the steady-state poly(A) tail-length distributions may not reflect deadenylation rates and may therefore not directly relate to mRNA half-lives. Unlike mRNA half-lives, however, transcriptional efficiency correlates with poly(A) tail length. Could the unexpected connection reflect a direct mechanistic link between transcription and polyadenylation? Transcription is integrated with mRNA processing, and polyadenylation requires interaction between Pol II and polyadenylation factors (Proudfoot et al., 2002). This raises the possibility that high transcription rates promote long poly(A) tails that in turn increase translational efficiency. However, our data on mRNAs expressed at different levels do not support this idea (Figure S9). They indicate that newly transcribed mRNAs contain long poly(A) tails that are deadenylated with different kinetics, and the final tail length is not influenced by the transcription rate. This view is consistent with data from budding yeast, which indicate that the 3′UTRs are critical to determine deadenylation rates and ultimate poly(A) tail lengths (T.H.B. and T.P., unpublished data).

The correlation between transcriptional and translational efficiency could reflect independent evolutionary selection for efficient expression of proteins in high demand at these two distinct levels of gene expression. In this scenario, the correlation between transcription and translation would not reflect any direct mechanistic link. Consistent with this view, the mRNA levels in our data (based on genes expressed from their native promoters) correlate with the protein levels from the ORFeome study ([Matsuyama et al., 2006] r = 0.23; p < 2e^−16^). This finding is striking given that the protein levels have been determined after expressing all genes from the same promoter, and the mRNA levels of the ORFeome study do not correlate with the protein levels (Matsuyama et al., 2006). Overall, evolutionary selection thus seems to independently but congruently influence both transcriptional and translational control to optimize gene expression for production of required protein levels.

Data from budding yeast suggest similar correlations between mRNA levels and ribosome density or ribosome occupancy, although these relationships have not been emphasized (Arava et al., 2003; Beilharz and Preiss, 2004; Beyer et al., 2004; Smirnova et al., 2005). Several groups have reported that mRNAs that become more highly transcribed in different conditions also become more efficiently translated (Preiss et al., 2003; Serikawa et al., 2003); this coordination between changes in transcription and changes in translation has been termed “potentiation.” The dynamics of deadenylation discussed above provides an explanation for the potentiation phenomenon. Increased transcription would temporarily increase the proportion of long-tailed mRNAs, which in turn would lead to increased translation. This could provide an elegant way for the cell to link changes in transcription with corresponding changes in translation on a global scale.

### Conclusions

Comparisons between our genome-wide data sets on key aspects of gene expression control, ranging from transcription to translation, highlight a remarkable degree of global interconnectivity between different layers of gene expression. The large network of correlations between all aspects of regulation suggests widespread coordination between multiple gene expression levels for coherent and efficient protein production. Some of these relationships may reflect direct mechanistic links (e.g., translational efficiency could influence mRNA stability), whereas others may reflect independent evolutionary selection at different levels of regulation (e.g., alignment of transcriptional and translational efficiencies). These rich data sets, all acquired under one standardized condition in a simple model organism, provide a framework to interpret global and specific regulation of gene expression in response to environmental or genetic perturbations, and they should advance mechanistic and systems-level insight into multilayered gene-expression programs.

## Experimental Procedures

### *S. pombe* Growth Condition

For all experiments, wild-type 972 *h^−^* cells were exponentially grown in Edinburgh minimal medium (EMM) at 32°C to a titer of ≤5 × 10^6^ cells/ml.

### Genome-Wide Translational Profiling

Polysome profiles were prepared essentially as previously described (Bachand et al., 2006). RNA isolation and microarray processing were as described by Arava et al. (2003) and Lyne et al. (2003), respectively. Normalization was based on spiked external RNA. Details of these methods can be found in the Supplemental Data. Translational profiling was performed in triplicate from independent biological repeats, including a dye swap. mRNAs fulfilling the following criteria were included for further analysis: (1) microarray data for all 12 fractions from at least two out of the three repeats were available, excluding 1012 mRNAs, and (2) the Pearson correlation between profiles for the same gene from the different repeats was ≥0.7 (if present in all three repeats) or ≥0.75 (if present in only two repeats), excluding 352 mRNAs.

Translation profiles were calculated as the percentage of a given mRNA for each fraction such that the total over all fractions was 100%. Ribosome occupancy for a given mRNA was calculated by adding up the percentages of this mRNA for ribosome-associated fractions 5–12. For the mean ribosome number bound to a given mRNA, we calculated the percentages of this mRNA for each ribosome-associated fraction such that the total of fractions 5–12 was 100%; the percentage of mRNA in each fraction was then multiplied with the corresponding estimate for associated ribosomes (see Supplemental Data), and these values were added up. Ribosome densities represent the mean ribosome number associated with each mRNA divided by the corresponding ORF length. All values were determined individually for each biological repeat and then averaged.

### PASTA Analysis and LM-PAT Assay of Poly(A) Tail-Length Distribution

Details of the fractionation of mRNAs based on poly(A) tail length followed by microarray interrogation (PASTA analysis) can be found in the Supplemental Data. RNA fractionation and microarray analysis were performed for two independent biological repeats with dye swap. Only mRNAs with data for all five fractions in both experiments were used for further analysis. In total, 2714 protein-coding mRNAs fulfilled these criteria of which 2575 were also included in the translational profiling data set. Ratios of each mRNA were transformed into percentages for each fraction. For relative ranking of poly(A) tail lengths, the percentages were multiplied by arbitrary weights of 0.1, 0.2, 0.3, 0.4, and 0.5 for fractions 1–5, respectively. LM-PAT assays were performed as described by Sallés and Strickland (1995). Details and primer sequences can be found in the Supplemental Data.

### Determination of mRNA Levels, Pol II Occupancy, and mRNA Half-Lives

mRNA steady-state levels were determined on Affymetrix Yeast 2.0 Genechip arrays by using standard methods specified by the supplier (see Supplemental Data). The signal intensities from two independent biological repeats were averaged, resulting in measurements for 4818 out of 4962 nuclear protein-coding genes.

ChIP of Pol II was performed with an antibody specific for the CTD domain (4H8, Upstate) and protein A Sepharose beads (GE Healthcare). The immunoprecipitated material and input DNA (reference) were labeled by using the Bioprime DNA labeling system (Invitrogen) and hybridized to microarrays (see Supplemental Data). Data are averages of two independent biological repeats, resulting in measurements for 4843 out of 4962 nuclear protein-coding genes.

To determine mRNA half-lives, transcription was blocked with 300 μg/ml 1,10-phenanthroline (Sigma) as described by Rodríguez-Gabriel et al. (2003), followed by microarray analysis (see Supplemental Data). Data from three independent time courses were used. Assuming exponential decay, a linear regression curve was fitted to the log ratios of each mRNA. The 868 mRNAs whose 95% confidence interval from the regression slopes did not include zero were categorized as “short half-lives,” whereas the 992 mRNAs with regression slopes closest to zero were categorized as “long half-lives.”

### Statistical Analyses

Spearman rank correlations (r) and corresponding p values were calculated with the cor.test function in the statistics package R (version 2.2.1). The weighted association map (Figure 7D, R-function gplot) was determined with all significant correlations (corrected for multiple testing and based on pair-wise complete cases). The edge widths were scaled by the absolute correlation values. P values for Figure 6 were calculated by using the two-sided Student's t test function in Microsoft Excel (assuming unequal variances). Enrichments for GO terms (Aslett and Wood, 2006) or functional lists were determined by using a test in GeneSpring (Agilent) based on the hypergeometric distribution. 391 genes were excluded from the ORFeome data set (Matsuyama et al., 2006), because their values for the relative protein expression level were zero.

### Access of Microarray Data

The data on all measured gene expression properties are provided in Table S1; all processed and normalized data sets are available from our website (http://www.sanger.ac.uk/PostGenomics/S_pombe/), and the entire raw data sets are available from ArrayExpress (see Accession Numbers).

## Accession Numbers

Accession numbers are as follows: E-TABM-222, translational profiling; E-TABM-220, mRNA levels; E-TABM-219, Pol II occupancy; E-TABM-218, mRNA half-lives; and E-TABM-217, PASTA analysis.

## Supplemental Data

Document S1. Supplemental Experimental Procedures, Supplemental References, and Ten Figures

Table S1. Processed Genome-Wide Data Sets on Ribosome Occupancy, Ribosome Number, Ribosome Density, ORF Length, Poly(A) Tail Rank, mRNA Level, Relative Pol II Occupancy, AugCAI, and mRNA Half-Life
