# Supplementary material for: A Network of Multiple Regulatory Layers Shapes Gene Expression in Fission Yeast
Source: Mol Cell. 2007 Apr 13;26(1):145–55. doi: 10.1016/j.molcel.2007.03.002 (PMC1885965; doi:10.1016/j.molcel.2007.03.002)
Supplement: Document S1. Supplemental Experimental Procedures, Supplemental References, and Ten Figures [file mmc1.pdf]

Molecular Cell, Volume *26*

Supplemental Data

A Network of Multiple Regulatory Layers

Shapes Gene Expression in Fission Yeast

Daniel H. Lackner, Traude H. Beilharz, Samuel Marguerat, Juan Mata, Stephen Watt, Falk Schubert, Thomas Preiss, and Jürg Bähler

Supplemental Experimental Procedures

Genome-wide translational profiling

Preparation of polysome profiles and microarray hybridization: Cycloheximide was added to a final concentration of 100 µg/ml 5 min before harvesting 50 OD_{600} of cells by centrifugation at 4ºC and washing in lysis buffer (Bachand and Silver, 2004). Cells were resuspended in 100 µl lysis buffer with 40 U/ml RNasin (Promega) and complete protease inhibitor cocktail (Roche). Cells were lysed in 1.5 ml tubes using a Fastprep cell disruptor with 1 g of chilled glass beads (BioSpec Products). After lysis, 400 µl of lysis buffer was added and a hole was poked in the bottom of the tube to separate the lysate by centrifugation into another 1.5 ml tube. The lysate was cleared by two centrifugations at 4ºC. Aliquots corresponding to 25 OD_{260} units in 350 µl were loaded onto an 11 ml linear 10-50% (w/v) sucrose gradient, which was prepared with the Gradient Master (Biocomp), and separated by centrifugation for 160 min at 35,000 rpm in a SW 40Ti rotor (Beckman). The gradients were then fractionated by upward displacement with 55% (w/v) sucrose, and fractions of ~900 µl were collected directly into tubes containing 2 ml of 8 M Guanidium-HCl using an Isco fractionation system. Corresponding fractions from 3 gradients were pooled and 5 in vitro-transcribed *Bacillus subtilis* mRNAs (Lyne et al., 2003) were added for normalization in the following amounts: Trp 0.09 ng/µl, Dap 0.45 ng/µl, Lys 0.9 ng/µl, Phe 4.5 ng/µl, and Thr 9 ng/µl. After addition of an equal volume of 100% ethanol, RNA from each fraction was precipitated overnight at -20ºC and centrifuged at 4ºC for 90 min. Further purification of the RNA by phenol:chloroform extraction and LiCl-precipitation was performed as described by Arava et al. (2003). RNA from each pellet was then resuspended in 20 µl of DEPC-treated H_2O. 10 µl of the RNA from each fraction was used for microarray analysis and labelled using oligo(dT) primers and the SuperScript™ Direct cDNA Labeling System (Invitrogen) and Cy3/Cy5-dCTP (Amersham). Test experiments including an additional random primer for RNA labelling (Lyne et al., 2003) showed that the oligo(dT) primers did not lead to any significant bias against mRNAs with short poly(A) tails, and our results were not affected by the primers (Figure S10). Polysomal RNA from each fraction was hybridized against 20 µg of total RNA extracted using the hot-phenol method (Lyne et al., 2003). Half the amount of bacterial mRNAs indicated above was added to each aliquot of total RNA before labeling. Hybridization to microarrays, slide-washing and scanning of microarrays was performed as described (Lyne et al., 2003).

Microarray data normalisation and processing: Data from translational profiling cannot be normalized on the assumption that the overall RNA levels from the competitive hybridization are similar between the two RNA samples. We therefore spiked bacterial mRNAs to normalize for different amounts of RNA in the individual fractions. Probes for the bacterial mRNAs on the microarray were first normalized locally to obtain an average signal ratio of 1. Normalization based on signal intensities was then done as described (Lyne et al., 2003). Fraction 10 in the 3rd repeat was a clear outlier showing signal intensities roughly twice as high as in the other 2 repeats and higher signals than its neighbouring fractions. Thus, all ratios for this fraction were divided by a correction factor, which was calculated based

on the best correlation to the other repeats. Data and conclusions presented here did not change if the 3rd experimental repeat was omitted, but the number of mRNAs included in the data-set was reduced to 3020.

Determination of ribosomes associated with each fraction: Associated ribosome numbers for each fraction were estimated by plotting the defined peaks from the polysome profile, representing 1 to 8 ribosomes, against the relative distance from the start of the profile and fitting an exponential curve; this curve was used to determine ribosome numbers at the beginning and end of a given fraction, which were averaged to produce the mean ribosome number associated with this fraction.

PASTA analysis of poly(A) tail length distribution

Poly(U) sepharose chromatography was based on published protocols (Binder et al., 1994) and product specifications (GE Healthcare cat# 17-0610-01), with the following modifications. Dry poly(U)-sepharose 4B was hydrated and washed 3x in [0.1 M NaCl; 10mM Tris-HCl pH7.4] at room temperature. Then followed washes in Elution Buffer (EB) [0.1 M NaCl; 0.01 M EDTA; 0.5 M Tris-HCl pH 7.4; 0.2% SDS; 25% formamide] and High Salt Binding Buffer (HSBB) [0.7 M NaCl; 0.01 M EDTA; 0.5 M Tris-HCl pH7.4; 0.2% lauryl sarcosine; 12% formamide], 1x each for 5 min at 70°C. Total cellular RNA was purified using the hot phenol method. For each run, 600 µl of HSBB, ~150 µl of wet gel volume, and 150 µg total yeast RNA were denatured at 70°C for 5 min in 1.5 ml tubes. For binding, tubes were incubated in an Eppendorf Thermomixer (1100 rpm in a cold room), first at 35°C for 10 min, followed by cooling to 12°C and for 90 min (including ~25 min ramp-down time). The matrix was washed 4x in batch with HSBB for 5 min at 12°C. Each thermal elution step was then performed in the Thermomixer, by re-suspending the matrix in 600 µl EB at the specified temperature for 5 min. The supernatant from a 1/2 min microcentrifuge spin at 6000 rpm containing the eluted mRNA was removed with an insulin syringe, re-spun and precipitated in 2 vol ethanol, 1/10 vol 5 M NaCl and coprecipitant (either glycogen or Pellet Paint from Novagen). Pelleted mRNA was resuspended in RNase-free dH2O, desalted using BIO-RAD Micro Bio-Spin6 chromatography columns, and dried. RNA from each fraction was resuspended in 10 µl of DEPC-treated H_2O and was used for labelling and subsequent microarray analysis as described above. Labelled RNA from each fraction corresponding to different elution temperatures was competitively hybridized against reference RNA eluted directly at the highest temperature, which contains mRNAs with the whole range of poly(A) tail lengths. Microarrays from each of the 5 fractions were normalized using our standard normalization script (Lyne et al., 2003). In this way, each mRNA was normalised against the global trend of poly(A) tail length distribution; an mRNA eluting in an average pattern will thus have a ratio of 1 for each fraction.

LM-PAT assay of poly(A) tail length distribution

LM-PAT assays were performed as described by Sallés and Strickland (1995) using as input 1 µg total RNA or 10% of each fraction eluted from poly(U) chromatography. Briefly, mRNA was incubated with oligo(dT)$_{12\text{-}18}$ primers (GE Healthcare) in the presence of T4-DNA ligase at 42°C followed by ligation of an oligo dT$_{12}$-anchor primer and further incubation at 12°C, thus covering the full length of poly(A) tails of mRNAs. cDNA was synthesised from the ligated primers with Superscript II (Invitrogen). Aliquots of this cDNA were then used in PCR reactions with Fast-Start polymerase (Roche) to amplify a region between a site in the 3' UTR of the mRNA under study and the 3' Anchor region. LM-PAT PCR products were visualised by 2% high-resolution agarose gel electrophoresis (Agarose 1000, Invitrogen) and scans on a FLA-5100 imager and MultiGauge software (Fujifilm). Some laddering of the products reflects a size bias in the oligo(dT)12-18 preparation. For bulk poly(A)-tail length analysis, 90% of each poly(U) chromatography fraction (or 1 µg total RNA) was 3' end-labelled with [32P]-pCp and digested with RNAses A and T1 as described (Minvielle-Sebastia et al., 1991). The remaining 32P-labelled poly(A) tracts were resolved by denaturing (urea) 16% PAGE and analysed by autoradiography or phosphorimaging using the FLA-5100 imager. The following primers were used:

PAT-Primer, GCGAGCTCCGCGGCCGCGTTTTTTTTTTTT
hhf1/2, GGCCGTACCATTTATGGTTTCGG
rpl14, GCTGTCGCTAAGGCTCTCAAGGCC
rps27, GCTCGTCTTATGGAGGGATGCTC
for3, GAGACATGTTGGGATAACGATTC
pom1, GCCGTGATCACACATTACCTTGG
rpb4, CTTGGATGAGCTTTCCACTTTGCG
urg1, CGGTCGTCATTGGTAAGCTTGCCC
SPAC1002.17c, GGTGATCGTTTGTATGGTGCCACAGC

Determination of steady-state mRNA levels

Hybridizations were performed using Affymetrix Yeast 2.0 Genechip arrays containing 5021 probe sets for 5031 predicted *S. pombe* genes. The *S. cerevisiae* probe sets on the same chip were masked out at the analysis stage. Target preparation was carried out using the standard Affymetrix Genechip eukaryotic hybridization protocols. Scanning was done on a Genechip Scanner 3000, and data extraction was carried out using Affymetrix GCOS 1.4. The software extracts the average signal intensities representing expression levels for each set of 11 probes/gene.

Determination of Pol II occupancy

Cells were crosslinked in 1% formaldehyde for 30 min at room temperature and frozen for later use. Pellets were washed in H_2O, resuspended in FA lysis buffer (50mM HEPES-KOH pH 7.6, 1mM EDTA pH 8, 150mM NaCl, 1% Triton X-100, 0.1% Na-deoxycholate) and lysed with glass beads in a FastPrep cell disruptor. The extracts were sonicated to fragment chromatin to an average size of ~500 bp with a Branson Digital Sonifier and cleared by centrifugation. The immunoprecipitation was performed immediately with an antibody specific for the Pol II CTD domain (4H8, Upstate) using protein A Sepharose beads (GE Healthcare). After washes and elution, samples were treated with proteinase K (Invitrogen) and the crosslink was reversed by overnight incubation at 65°C. DNA was purified by phenol/chloroform extraction, precipitated, and treated with RNase. After purification on QIAquick columns (Qiagen) the immunoprecipitated material and input DNA (reference) were labelled using the Bioprime® DNA labelling system (Invitrogen) and hybridised to DNA microarrays as described for translational profiling.

Determination of mRNA half-lives

Cells were treated with 300μg/ml 1,10-phenanthroline, 6μg/ml 6-Azauracil, or 2.5mg/ml Thiolutin (all Sigma) to block transcription. Cells samples were harvested and immediately frozen, before, or 4, 12 and 28 min after addition of the drug. RNA was isolated by phenol extraction, and purified with the RNeasy kit (Qiagen). RNA labelling was performed using random and oligo(dT) primers in the presence of bacterial spikes (Lyne et al., 2003). Samples from time points 4, 12, and 28 min where hybridised on DNA microarrays using time point 0 as a reference. Microarrays were normalised using bacterial spikes as described by Lyne et al. (2003). Further analyses revealed that treatments with 6-Azauracil and Thiolutin triggered a strong stress response in fission yeast, while cells treated with 1,10-phenanthroline showed little or no stress response. We therefore chose 1,10-phenanthroline to estimate mRNA half-lives.

Figure S1. Distribution of mRNA levels for protein-coding genes included or excluded from the translational profiling data

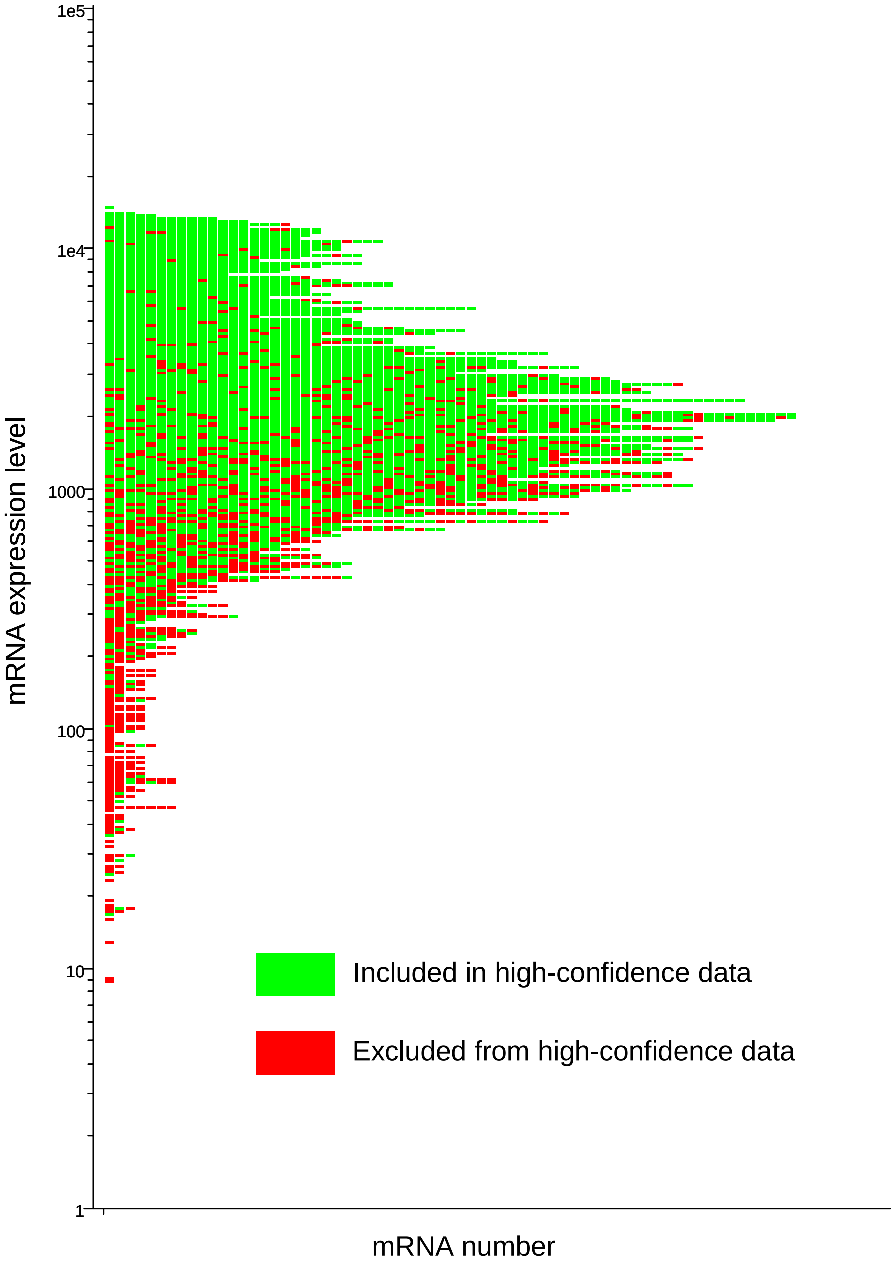

Histogram showing the mRNA levels for 4818 protein-coding genes that provided signal data on Affymetrix chips (Experimental Procedures). The average of two independent experiments is shown. Green: genes included in the high-confidence data set from the translational profiling experiments (3567 genes with measurable chip signals). Red: genes not included in the high-confidence data set (1251 genes with measurable chip signals).

Figure S2. Correlation between ORF length and mean number of associated ribosomes

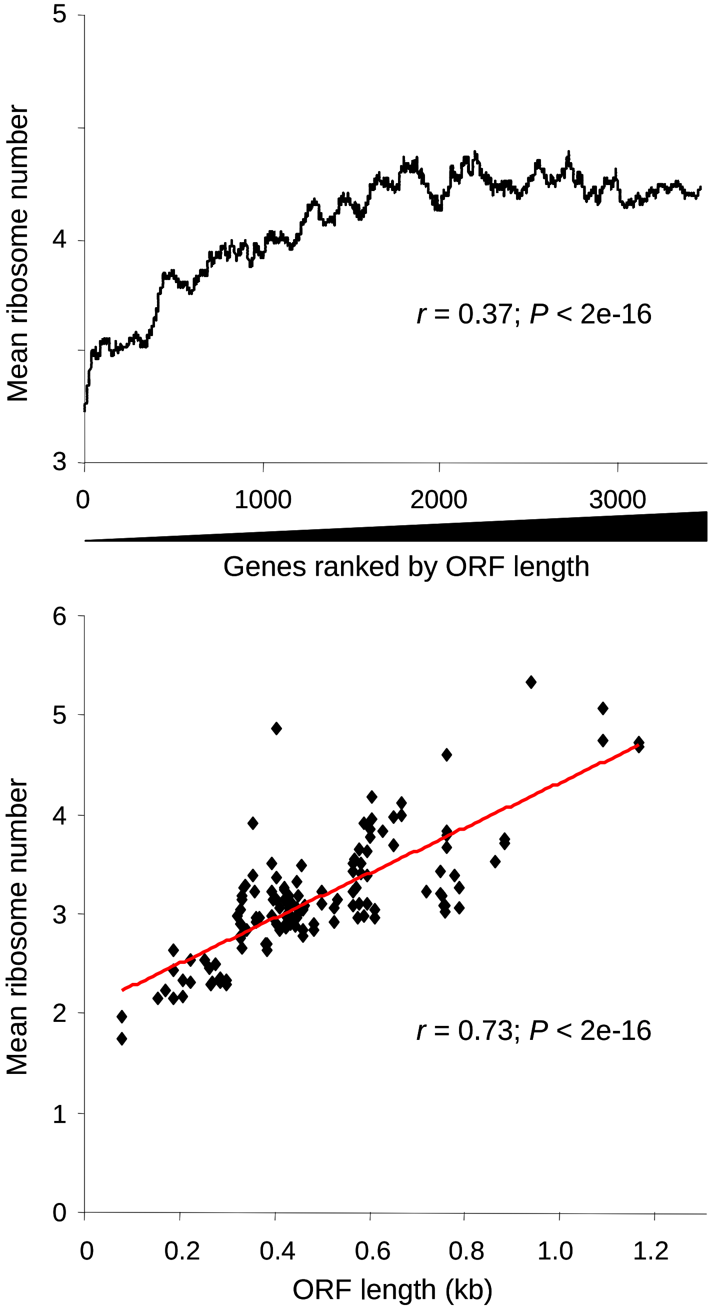

Top: Graph showing moving averages (100-gene window) of mean ribosome number as a function of genes ranked by ORF length (n = 3598), along with the corresponding Spearman rank correlation.

Bottom: Scatter plot of ORF length against mean number of associated ribosomes for mRNAs encoding ribosomal proteins (n = 134). The red line represents the linear trend-line for this correlation. The corresponding Spearman rank correlation between ORF length and mean ribosome number is also shown.

Figure S3. Correlations of AugCAI values with mRNA features

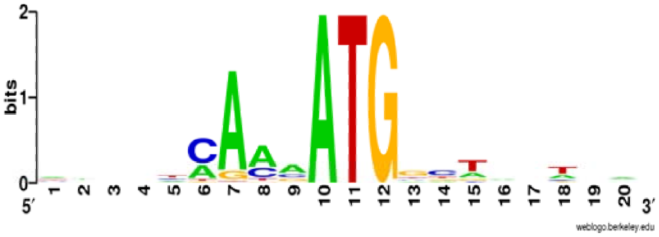

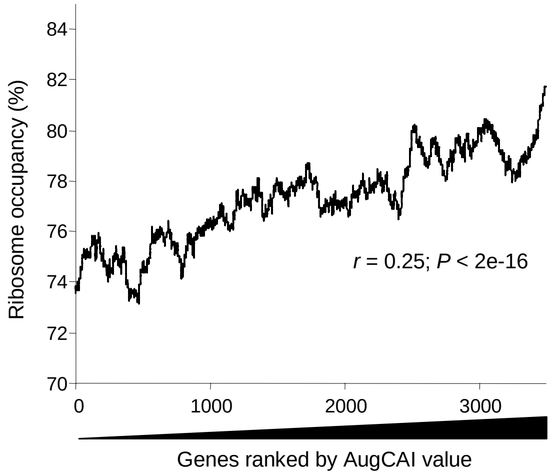

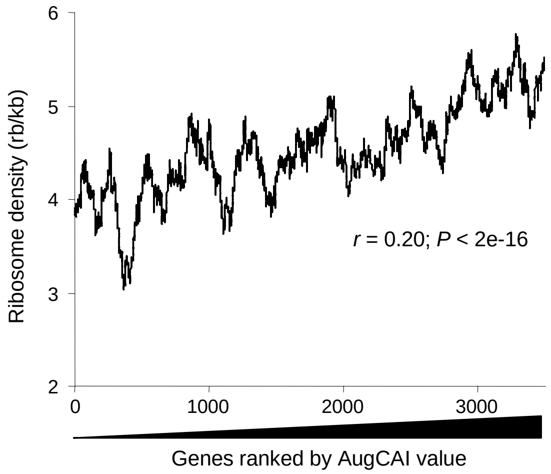

Top: Consensus sequence for optimal translation initiation derived from the 100 most abundant mRNAs. The WebLogo tool was used for visualization (weblogo.berkeley.edu). Based on this consensus sequence, AugCAI values were calculated for all mRNAs as described by Miyasaka (1999). An almost identical motif was derived from the 100 mRNAs with highest ribosome density (not shown).
Middle: Graph showing moving averages (100-gene window) of ribosome occupancy as a function of genes ranked by AugCAI values (n = 3593), along with the corresponding Spearman rank correlation.
Bottom: Graph showing moving averages (100-gene window) of ribosome density as a function of genes ranked by AugCAI values (n = 3593), along with the corresponding Spearman rank correlation.

Figure S4. Overestimation of ribosome number for fraction 12 does not affect negative correlation between ribosome density and ORF length

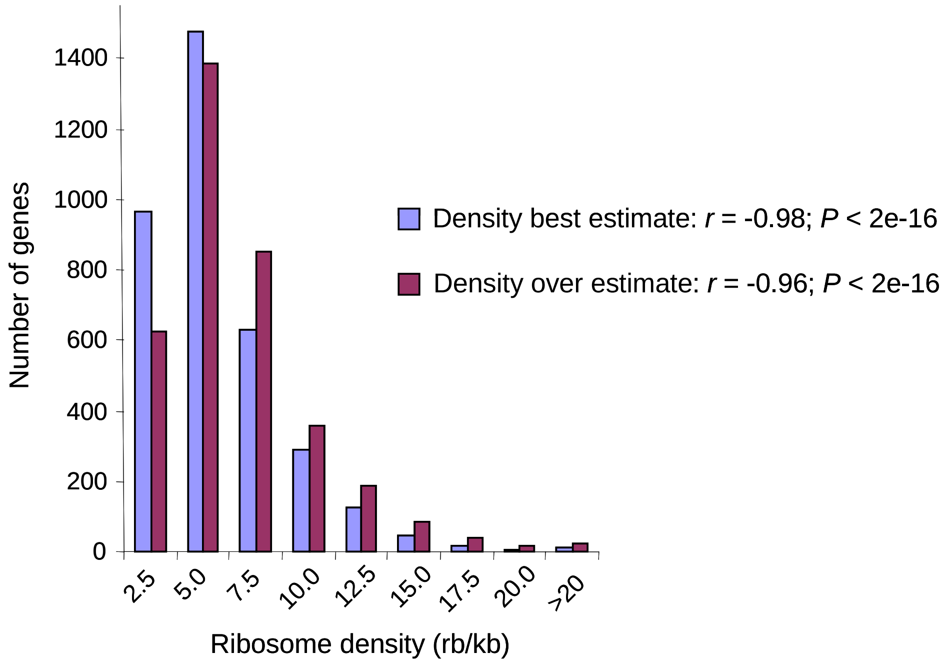

Histogram using bins of different ribosome densities (upper bin limits given on X axis). Blue: distribution of ribosome densities calculated as described in Experimental Procedures. Purple: distribution of ribosome densities calculated the same way, except that the number of ribosomes associated with mRNAs in fraction 12 were 2-fold overestimated. The corresponding Spearman rank correlations between ORF length and ribosome densities calculated in both ways are also shown.

Figure S5. Fractionation of mRNAs by poly(A)-tail length

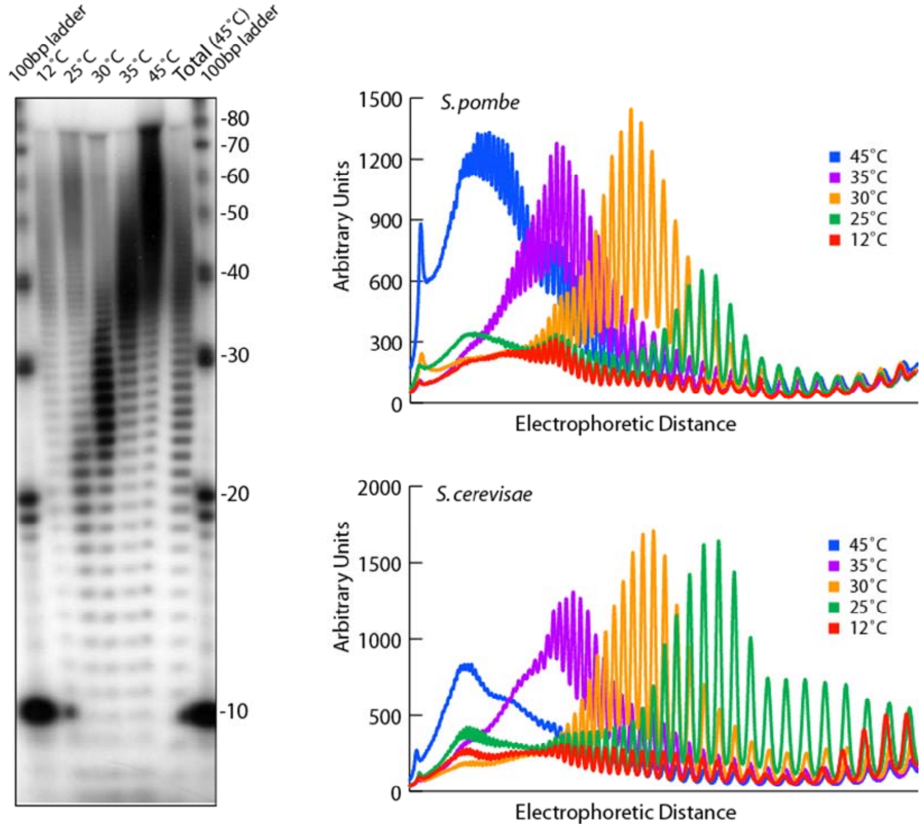

Left: Gel of poly(A) tail length tracts for mRNAs eluted from a poly(U) sepharose column at increasing temperatures as indicated on top, showing mRNAs with increasing poly(A) tail length from *S. pombe*. Nucleotide numbers corresponding to the 100 bp ladder are indicated at right. To obtain unfractionated mRNA reference material, all bound material was eluted in one batch at 45°C. There was minor cross-contamination of long-tailed mRNAs in the first two elution fractions; these transcripts may have bound non-specifically to the matrix or through poly(A) runs within the body of the transcript. Note also that short A-tract fragments are inefficiently precipitated by ethanol in the bulk end-labelling experiment, hence they show up relatively weakly.

Right: Graphs of poly(A) tail length tracts for *S. pombe* and *S. cerevisiae* showing relative intensity on the gel as a function of electrophoretic distance for each of the fractions. Each elution is enriched for a distinct population of poly(A) tail lengths (peak sizes for *S. pombe* in nucleotides: 12°C, ~10; 25°C, ~22; 30°C, ~30; 35°C, ~40; and 45°C, ~57).

Figure S6. Verification of PASTA analysis by LM-PAT assays

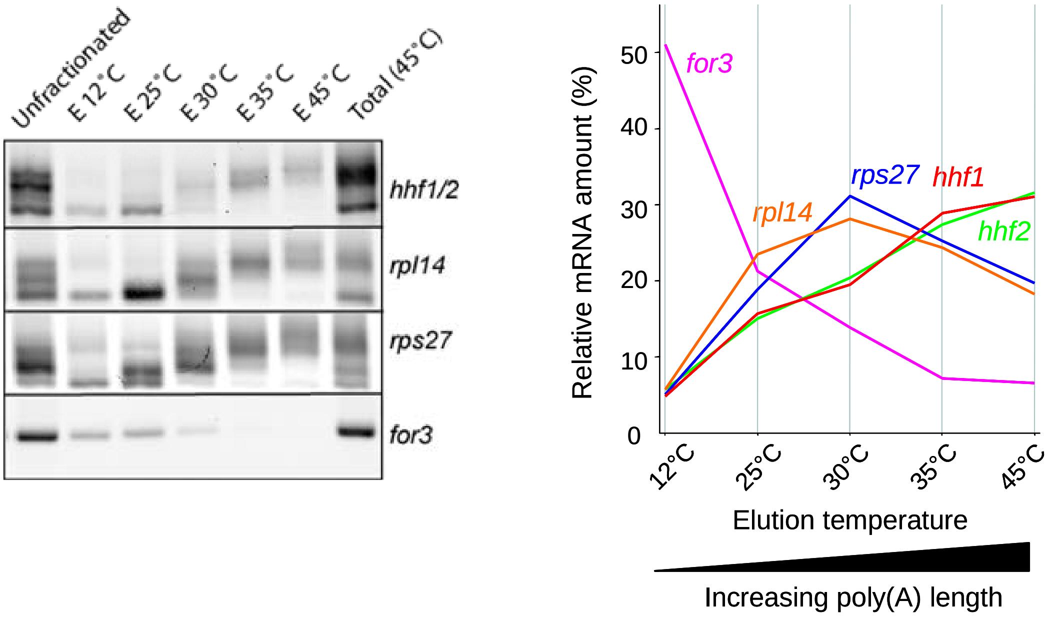

Left: Distribution of poly(A) tail length determined using LM-PAT assays. Fractions eluted from poly(U) sepharose column at increasing temperatures (corresponding to increasing tail lengths) as indicated on top. The following mRNAs were tested: *hhf1* and *hhf2* (almost identical mRNAs encoding histones, enriched for long poly(A) tails), *rpl14* and *rps27* (mRNAs encoding ribosomal proteins, enriched for poly(A) tails of medium length), and *for3* (mRNA encoding formin, enriched for short poly(A) tails).
Right: Profiles of poly(A) tail length distribution for the same mRNAs as determined by microarray-based PASTA analysis. The curves show the relative amounts of RNA for a given mRNA in each of the five fractions. Different mRNAs are color-coded. There is good agreement between poly(A) tail length distributions determined by the two independent approaches.

Figure S7. ORF length and mRNA levels do not correlate

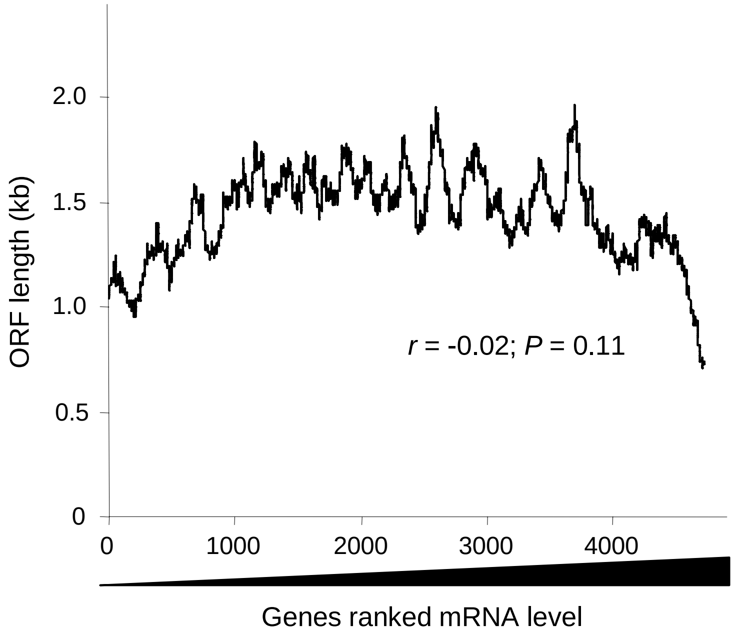

Transcript length vs mRNA level: *r* = -0.06; *P* = 0.41

Graph showing moving averages (100-gene window) of ORF length as a function of genes ranked by mRNA level (n = 4818). The corresponding Spearman rank correlation between ORF length and mRNA level is shown within the graph. The Spearman rank correlation between transcript length and mRNA level is also shown at the bottom. This was calculated using the 198 mRNAs for which 5'- and 3'-UTR length data are available in *S. pombe* GeneDB (www.genedb.org/genedb/pombe/index.jsp).

Figure S8. Decay of mRNAs with short and long half-lives

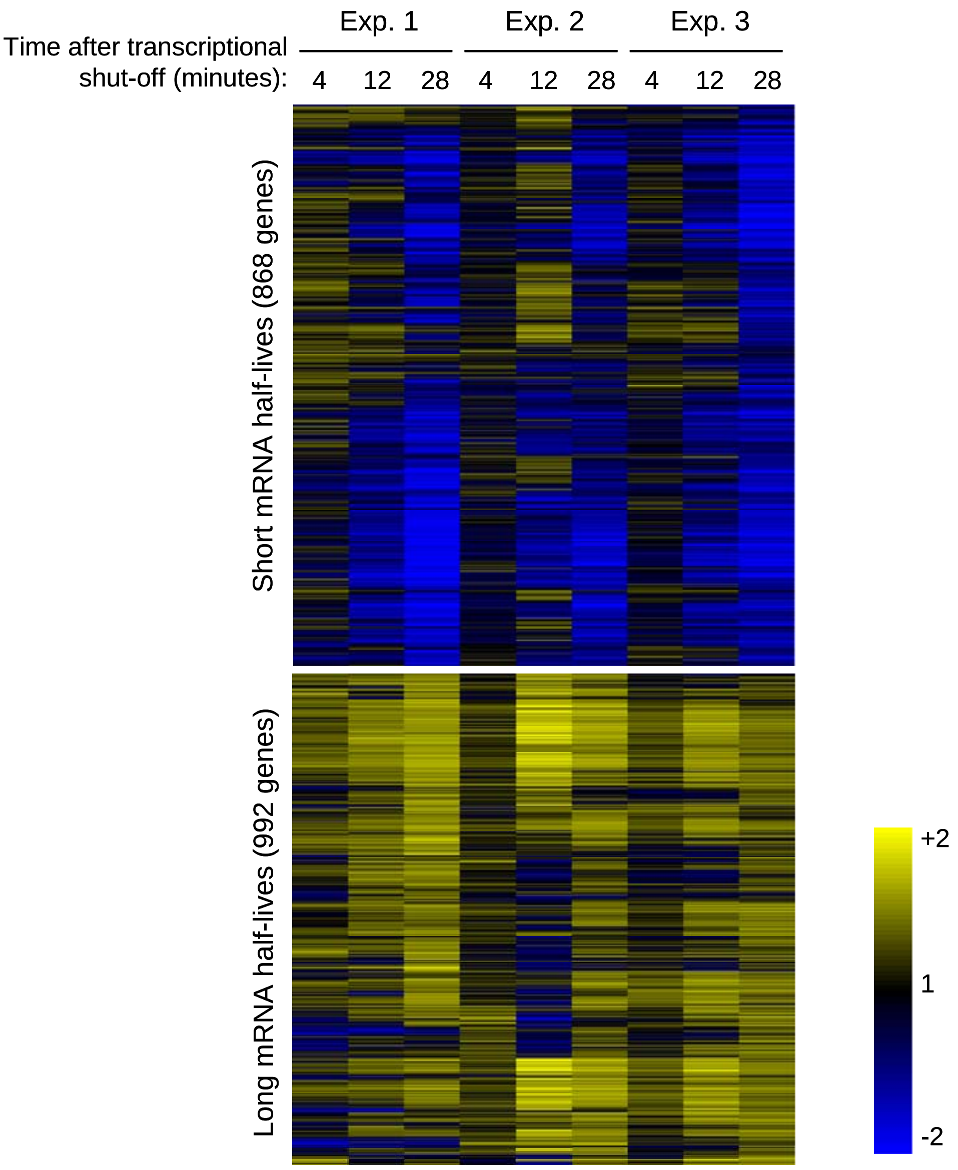

To estimate mRNA half-lives, cells were treated with the transcriptional inhibitor phenanthroline, and mRNA was isolated before and at 4, 12, and 28 min after transcriptional shut-off. Two lists of genes with short and long half-lives were created from these data (Experimental Procedures). The figure shows heat maps of these two gene lists, clustered using the Spearman correlation. Data from three independent biological experiments are shown. The columns represent experimental timepoints, and rows represent genes. The data of each array were normalized to the 50th percentile of the measurements taken from that array and color-coded according to the ratios between experimental samples vs sample before transcriptional shut-off.

Figure S9. Changes in transcription and poly(A) tail length

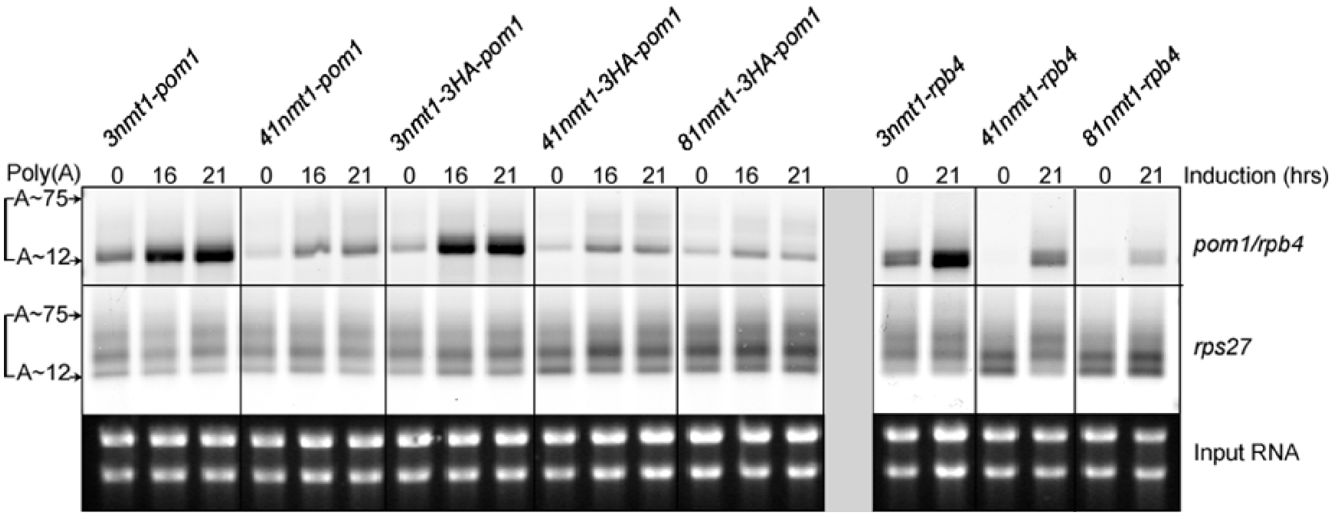

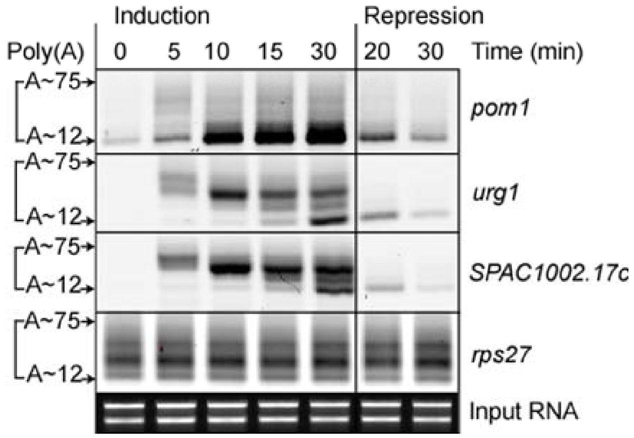

Top: The *pom1* (left panels; Bähler and Nurse, 2001) and *rpb4* (right panels; Sharma et al., 2006) genes were transcriptionally induced by thiamine removal using regulatable *nmt1* promoters of different strength: *3nmt1*, strongest promoter; *41nmt1*, intermediate promoter; and *81nmt1*, weakest promoter (Basi et al., 1993). mRNAs before induction (0) as well as 16 and 21 hours after induction were analysed for poly(A) tail length by LM-PAT assays. Both mRNAs showed short poly(A) tails independently of transcription rates. The longer-tailed *rps27* mRNA is included as a control (middle panels), and the input RNA is shown below.
Bottom: Transcriptional induction of *pom1* under the control of the regulatable *urg1* promoter showing a fast induction time (S. Watt, J. Mata, G. Burns, and J. Bähler, manuscript in preparation). A 30-min timecourse of induction was followed by a 30-min timecourse of repression 4 hours later, and mRNAs were analysed for poly(A) tail length by LM-PAT assays. Long-tailed forms of *pom1* mRNA are present at 5 min after induction, but from 10 min onwards the short-tailed form predominates. Corresponding LM-PAT assays from the same cells are also shown for *urg1* (under its own promoter) and SPAC1002.17c (which shows a similar short induction time under its own promoter). These mRNAs have slower deadenylation kinetics, and long-tailed forms are still evident 30 min after induction, but are gone at the time of repression. The unregulated *rps27* mRNA and input RNA are shown as controls.

Figure S10. Labelling with oligo(dT) primers does not create bias

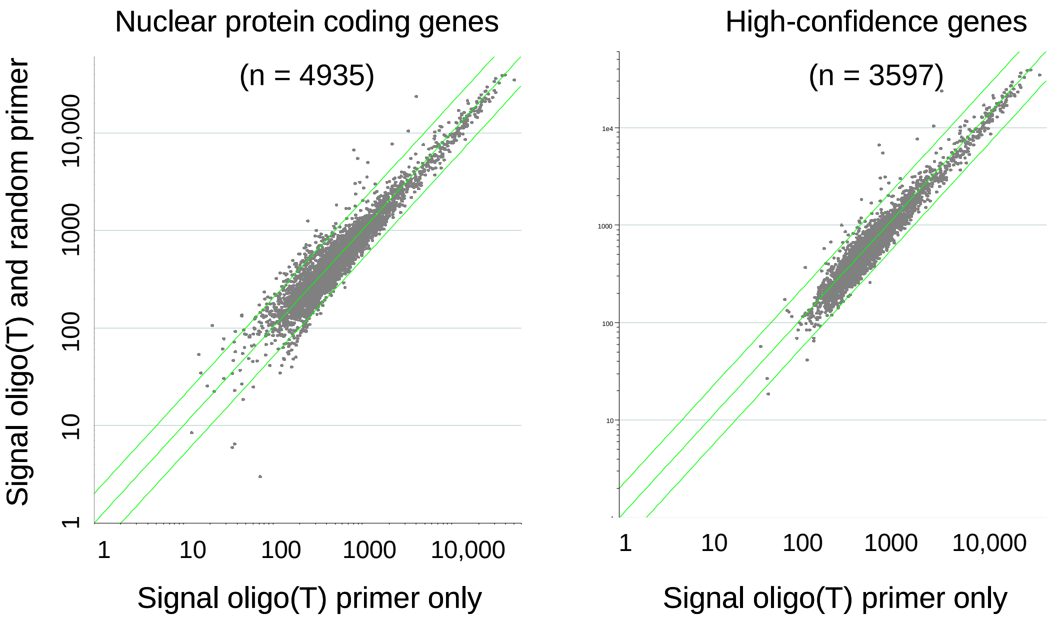

To address whether the use of oligo(dT) primers to generate cDNA could lead to a potential bias against mRNAs with short poly(A) tails, we performed four additional microarray hybridizations. We labelled different RNA pools with a mixture of random and oligo(dT) primers and hybridized these samples directly against the same RNA pools labelled only with oligo(dT) primers. Only 66 to 233 genes out of the 5165 genes present on the arrays showed a >2-fold difference in signals between the two labelling methods (~1.3%-4.5% of all genes). Moreover, most of these genes were lowly expressed and therefore showed inherently variable and unreliable signals. When considering only the high-confidence genes that were actually used in our analysis, only 17 to 70 genes were differentially expressed (~0.5%-2.6%). One of these four comparisons is shown in the figure (using an RNA pool of fractions 1-4 from translational profiling experiment). Assuming a worst case scenario that all the differentially expressed genes were badly labelled by the oligo(dT) primer, we recalculated the correlations between the poly(A) profiling data and the other data sets. The 76 genes that showed a >2-fold difference in signals between the two labelling methods but had been lost in one repeat due to poor labelling were now also included in the analysis. All the correlations were robust and did not change the conclusions. The biggest resulting change was that the correlation coefficient between mRNA levels and poly(A) tails increased from 0.46 to 0.49.
Note that even the few genes that are differentially labelled due to primer differences are in fact not expected to lead to any bias: if they are weakly labelled with oligo(dT) primers, they would also be weakly labelled in the reference sample, and any relative differences to other mRNAs would be normalized away. Since we do not work with absolute mRNA levels but with ratios relative to a reference, the shape and peaks of the translational and poly(A) profiles are not affected even with some mRNAs being more weakly labelled. Due to this robust 2-color reference approach, one would not expect that a relative under-representation of mRNAs with short poly(A) tails would change our results.

Supplemental References

Arava, Y., Wang, Y., Storey, J.D., Liu, C.L., Brown, P.O., and Herschlag, D. (2003). Genome-wide analysis of mRNA translation profiles in *Saccharomyces cerevisiae*. Proc. Natl. Acad. Sci. USA *100*, 3889-3894.

Bachand, F., and Silver, P.A. (2004). PRMT3 is a ribosomal protein methyltransferase that affects the cellular levels of ribosomal subunits. EMBO J. *23*, 2641-2650.

Bähler, J. and Nurse, P. (2001). Fission yeast Pom1p kinase activity is cell cycle regulated and essential for cellular symmetry during growth and division. EMBO J. *20*, 1064-1073.

Basi, G., Schmid, E., and Maundrell, K. (1993). TATA box mutations in the *Schizosaccharomyces pombe nmt1* promoter affect transcription efficiency but not the transcription start point or thiamine repressibility. Gene *123*, 131-136.

Binder, R., Horowitz, J.A., Basilion, J.P., Koeller, D.M., Klausner, R.D., and Harford, J.B. (1994). Evidence that the pathway of transferrin receptor mRNA degradation involves an endonucleolytic cleavage within the 3' UTR and does not involve poly(A) tail shortening. EMBO J *13*, 1969-1980.

Lyne, R., Burns, G., Mata, J., Penkett, C.J., Rustici, G., Chen, D., Langford, C., Vetrie, D., and Bähler, J. (2003). Whole-genome microarrays of fission yeast: characteristics, accuracy, reproducibility, and processing of array data. BMC Genomics *4*, 27.

Miyasaka, H. (1999). The positive relationship between codon usage bias and translation initiation AUG context in Saccharomyces cerevisiae. Yeast *15*, 633-637.

Minvielle-Sebastia, L., Winsor, B., Bonneaud, N., and Lacroute, F. (1991). Mutations in the yeast RNA14 and RNA15 genes result in an abnormal mRNA decay rate; sequence analysis reveals an RNA-binding domain in the RNA15 protein. Mol Cell Biol *11*, 3075-3087.

Sallés, F.J., and Strickland, S. (1995). Rapid and sensitive analysis of mRNA polyadenylation states by PCR. PCR Methods Appl. *4*, 317-321.

Sharma, N., Marguerat, S., Mehta, S., Watt, S., and Bähler, J. (2006). The fission yeast Rpb4 subunit of RNA polymerase II plays a specialized role in cell separation. Mol. Genet. Genomics *276*, 545-554.
